# A PDZ-Like Motif in the Biliary Transporter ABCB4 Interacts with the Scaffold Protein EBP50 and Regulates ABCB4 Cell Surface Expression

**DOI:** 10.1371/journal.pone.0146962

**Published:** 2016-01-20

**Authors:** Quitterie Venot, Jean-Louis Delaunay, Laura Fouassier, Danièle Delautier, Thomas Falguières, Chantal Housset, Michèle Maurice, Tounsia Aït-Slimane

**Affiliations:** 1 Sorbonne Universités, UPMC Univ Paris 06, INSERM, UMR_S 938, Centre de Recherche Saint-Antoine, Paris, France; 2 Assistance Publique-Hôpitaux de Paris, Hôpital Saint-Antoine, Centre de Référence Maladies Rares Maladies Inflammatoires des Voies Biliaires & Service d’Hépatologie, Paris, France; Hungarian Academy of Sciences, HUNGARY

## Abstract

ABCB4/MDR3, a member of the ABC superfamily, is an ATP-dependent phosphatidylcholine translocator expressed at the canalicular membrane of hepatocytes. Defects in the *ABCB4* gene are associated with rare biliary diseases. It is essential to understand the mechanisms of its canalicular membrane expression in particular for the development of new therapies. The stability of several ABC transporters is regulated through their binding to PDZ (PSD95/DglA/ZO-1) domain-containing proteins. ABCB4 protein ends by the sequence glutamine-asparagine-leucine (QNL), which shows some similarity to PDZ-binding motifs. The aim of our study was to assess the potential role of the QNL motif on the surface expression of ABCB4 and to determine if PDZ domain-containing proteins are involved. We found that truncation of the QNL motif decreased the stability of ABCB4 in HepG2-transfected cells. The deleted mutant ABCB4-ΔQNL also displayed accelerated endocytosis. EBP50, a PDZ protein highly expressed in the liver, strongly colocalized and coimmunoprecipitated with ABCB4, and this interaction required the QNL motif. Down-regulation of EBP50 by siRNA or by expression of an EBP50 dominant-negative mutant caused a significant decrease in the level of ABCB4 protein expression, and in the amount of ABCB4 localized at the canalicular membrane. Interaction of ABCB4 with EBP50 through its PDZ-like motif plays a critical role in the regulation of ABCB4 expression and stability at the canalicular plasma membrane.

## Introduction

The superfamily of ABC (ATP-binding cassette) transporters comprises a large number of membrane proteins, which mediate the translocation of a wide variety of molecules across cellular membranes. ABCB4, also called MDR3 (multidrug resistance 3) is a transporter expressed at the canalicular membrane of hepatocytes, where it translocates phosphatidylcholine (PC) into bile [1, 2]. In the aqueous environment of bile, phospholipids form mixed micelles with cholesterol and bile acids, thereby preventing the formation of cholesterol gallstones and the detergent action of free bile acids [3, 4]. Pathogenic mutations in the *ABCB4* gene sequence are associated with rare biliary diseases, in particular progressive familial intrahepatic cholestasis type 3 (PFIC3), which develops early in childhood and may be lethal in the absence of liver transplantation [5–7]. Perspectives to treat PFIC3 patients by pharmacological means have been recently opened with the observation that cyclosporin A was able to partially rescue an ABCB4 misfolded mutant retained in the endoplasmic reticulum [8]. However, rescued mutants may remain conformationally unstable after having reached their proper localization [9]. Therefore, therapeutic efforts to correct a folding defect must also aim at strengthening the stability of the mutant protein at the plasma membrane.

The mechanisms that control the stability of ABCB4 at the canalicular membrane are poorly known. PDZ (post-synaptic density 95/disks large/zonula occludens-1) domain- containing proteins act as scaffolds by linking transmembrane proteins to the cytoskeleton, and thus regulate their subcellular localization, activity, stability and mobility in the membrane [10, 11]. PDZ protein NHERF-1 (sodium-hydrogen exchanger regulatory factor-1), also known as EBP50 (ezrin-radixin-moesin (ERM)-binding phosphoprotein 50) is highly expressed in the liver, at the apical membrane of biliary epithelial cells, and at the canalicular membrane of hepatocytes [12, 13] and has been shown to control the membrane localization, stability and function of the ABC transporters ABCC7/CFTR and ABCC2/MRP2 [14, 15]. EBP50 is a multifunctional scaffolding protein, with two PDZ-domains at its N-terminus and a C-terminal domain that binds the ERM family of cytoskeletal proteins [16]. PDZ domains comprise 70–90 amino acids that bind preferentially to short sequences at the C-termini of target proteins [17]. They are grouped into three classes based on the target sequence. Class I PDZ domains recognizes the sequence motif -x-[S/T]-x-Φ, where x represents any residue, and ϕ a hydrophobic residue [18]. Class II recognizes the consensus motif (-x-ϕ-x-ϕ) [19], whereas class III prefers negatively charged amino acid at the -2 position and recognizes the consensus motif -x-[D/E]-x-ϕ [20].

The C-terminal regions of ABCB4 and of the drug transporter ABCB1/MDR1 are conserved, except for the last three amino acids. ABCB4 ends by the sequence glutamine-asparagine-leucine (QNL), while the last three amino acids of ABCB1 are lysine-arginine-glutamine (KRQ). Although the QNL motif of ABCB4 does not perfectly match any of the three classes of PDZ binding motifs, the presence of a hydrophobic amino acid at the extreme C-terminus suggests properties of a PDZ-binding-like motif. The aim of the work was to study the role of the QNL motif, and its potential binding to the PDZ protein EBP50. Studies were performed in the polarized hepatoma cell line HepG2, stably expressing wild type ABCB4 (ABCB4-wt) or the QNL-deleted mutant (ABCB4-ΔQNL). We show that deletion of the QNL motif decreased ABCB4 stability and accelerated its endocytosis. ABCB4 and EBP50 coimmunoprecipitated, and this interaction required the QNL motif. Moreover, down-regulation of EBP50 by small interfering RNA (siRNA) or by expression of an EBP50 dominant-negative mutant caused a significant decrease in the level of ABCB4 protein expression and in the amount of ABCB4 localized at the canalicular membrane. Our results thus identify EBP50 as a binding partner of ABCB4 and highlight a critical role for the PDZ-like motif in the expression and stability of ABCB4 at the canalicular membrane.

## Materials and Methods

### Antibodies and Reagents

The monoclonal P3II-26 anti-ABCB4 antibody was obtained from Enzo Life Science (Villeurbanne, France). Rabbit polyclonal anti-EBP50 was from Thermo Fisher Scientific (Rockford, IL). Rabbit polyclonal anti-Flag was obtained from Abcam (Cambridge, UK) and the monoclonal anti-β actin from Sigma-Aldrich (Saint-Quentin-Fallavier, France). Rabbit polyclonal anti-c-myc was obtained from Santa Cruz Biotechnology (Santa Cruz, CA). Alexa Fluor-labeled secondary antibodies, DRAQ5 fluorescent probe and culture media were from Invitrogen-Life Technologies (Cergy-Pontoise, France) and peroxidase-conjugated secondary antibodies were from Rockland Immunochemicals (Gilbersville, PA). The siRNA-EBP50 duplex, the control siRNA and the ECL-Plus detection kit were from GE Healthcare France (Orsay, France). The transfection reagents Turbofect and Fugene HD were purchased from Fermentas France (Villebon-sur-Yvette, France) and Promega (Charbonnières, France), respectively. All other reagents were obtained from Sigma-Aldrich.

### DNA Constructs, Mutagenesis and Polymerase Chain Reaction

The construction of the human wild type ABCB4 (ABCB4-wt), isoform A in the pcDNA3 vector has been previously described [21]. To produce the ABCB4-ΔQNL mutant, site directed mutagenesis was performed using the QuikChange II XL-mutagenesis kit from Agilent Technologies (Massy, France). DNA primers used were from Eurogentec (Angers, France): 5’-CCAGGCTGGGACACAGAACTTATGACTCGAGCGGCCG -3’ (sense) and 5’-CGG CCGCTCGAGTCATAAGTTCTGTGTCCCAGCCTGG -3’ (antisense). A triple c-myc tag with linkers (GEQKLISEEDLNGEQKLISEEDLNGEQKLISEEDLNG) was inserted in the first extracellular loop of ABCB4 between Ser99 and Leu100 (3xmyc-ABCB4-wt) by Genscript (Piscataway, NJ). The later template was used to generate the truncated ΔQNL ABCB4 (3xmyc-ABCB4-ΔQNL) using the same primers as above. The constructions of Flag-tagged full-length EBP50 and Flag-tagged PDZ1+PDZ2 were previously described [22]. All constructs were verified by automated sequencing.

### Cell Culture and Transfection

Hepatocellular carcinoma, human (HepG2) cells and human embryonic kidney (HEK) 293 cells were grown at 37°C in Dulbecco’s modified Eagles medium (DMEM) as previously reported [23]. HepG2 cells did not express detectable endogenous ABCB4 (data not shown). Human hepatocytes were isolated from surgical liver samples according to previously described methods [24], upon agreement (L 1232–3) from the Biomedicine Agency.

To stably express ABCB4-wt and ABCB4-ΔQNL in HepG2 cells, we used the pMSCV viral vector as the expression system (Clontech, Basingstoke, UK). Human ABCB4 cDNA was amplified and inserted between *Eco*RI and *Bgl* II restriction sites of the pMSCV-neo plasmid. Corresponding vectors pMSCV-ABCB4-wt and pMSCV-ABCB4-ΔQNL plasmids were purified (Promega). GP2-293 packaging cells (4.2x10^6^) plated in 100 mm Petri dishes were cotransfected with 6 μg of pMSCV constructs and 6 μg of pAmpho (encoding for amphotropic envelope protein) using Fugene HD at reagent:DNA ratio of 3:1. Twenty-four hours post-transfection, media from packaging cells were collected and centrifuged at 800 rpm for 10 minutes. The supernatant was then filtered through a 0.45-μm cellulose acetate and supplemented with polybrene (8 μg/mL). HepG2 cells (10 ^6^) were infected by incubation with the virus preparation for 36 hours. For selection of stably transfected HepG2 cells, cells were grown for 10 days in the presence of neomycine at 800 μg/mL.

Transient transfections were performed with cDNAs encoding 3xmyc-ABCB4-wt and 3xmyc-ABCB4-ΔQNL constructs in HEK 293 cells and with cDNAs encoding EBP50-Flag and PDZ1+PDZ2-Flag constructs in ABCB4-wt- and ABCB4-ΔQNL-expressing HepG2 cells using the Turbofect transfection reagent following the manufacturer’s instructions.

### Immunofluorescence and Confocal Microscopy

ABCB4-wt- and ABCB4-ΔQNL-expressing HepG2 cells were fixed with methanol/acetone (4:1, v/v) at -20°C. Incubations with primary and secondary antibodies were performed as previously described [8]. Confocal imaging was acquired with a Leica TCS SP2 Laser Scanning Spectral system attached to a Leica DMR inverted microscope. Optical sections were recorded with a 63/1.4 oil immersion objective. Laser scanning confocal images were collected, and analyzed using the on-line “Scan Ware” software. Images were processed using ImageJ 1.41 and Photoshop softwares. Figure compilation was accomplished using Adobe Photoshop 5.5 and Adobe Illustrator 10.

### Analysis of ABCB4 Protein Stability

ABCB4-wt- and ABCB4-ΔQNL-expressing HepG2 cells were incubated with cycloheximide (25 μg/mL) to inhibit further protein synthesis. Following incubation for different time periods, cells were harvested, lysed and subjected to SDS-PAGE and immunoblot analysis. Band density was quantified by the ImageJ 1.41 software and normalized so that the density at time point 0 was 100%. The amounts of remaining ABCB4 were expressed as a percentage relative to point 0.

### Internalization Assay

Internalization assay was performed according to a previously published protocol [25]. In brief, transiently transfected HEK 293 cells were washed three times with HEPES-buffered (20 mmol/L, pH 7.0) serum-free medium (HSFM). Cell surface antigens were labeled at 0°C for 30 minutes with anti-myc antibody, diluted in HSFM/0.2% BSA. After surface labeling, cells were extensively washed with HSFM/0.2% BSA, placed in prewarmed complete medium, and incubated at 37°C for 60 minutes. Non-internalized antibody-antigen complexes were removed by acid washing (200 mmol/L glycine, 150 mmol/L NaCl, pH 2.5) before fixation, permeabilization and staining with fluorescently labeled secondary antibodies. Fluorescence was examined by confocal microscopy.

### Coimmunoprecipitation and Western Blotting

Coimmunoprecipitation of ABCB4 and EBP50 was performed using the Pierce Coimmunoprecipitation Kit (Thermo Fisher Scientific) and following the manufacturer’s instructions. Briefly, ABCB4-wt- and ABCB4-ΔQNL-expressing HepG2 cells and freshly isolated human hepatocytes were washed with phosphate-buffered saline (PBS) and lysed at 4°C in lysis buffer containing 25 mmol/L Tris, pH 7.4, 150 mmol/L NaCl, 1 mmol/L EDTA, 1% NP-40, 5% glycerol, and the cell lysates were cleared by centrifugation at 10,000 x g for 10 minutes at 4°C. Protein concentration was determined by Uptima bicinchoninic acid protein assay (Interchim, Montluçon, France). Immunoprecipitation was performed overnight at 4°C with 1 mg of protein lysate and 4 μg of anti-ABCB4 or 4 μg of immunoglobulins from normal mouse serum (produced in our laboratory) crosslinked to protein A/G agarose beads by disuccinimidyl suberate (DSS) for 1 hour at room temperature. Immunoprecipitated proteins were subjected to immunoblotting with ABCB4 and EBP50.

### RNA Interference

ABCB4-wt- and ABCB4-ΔQNL-expressing HepG2 cells were transfected with 100 nmole/L EBP50 siRNA or 100 nmole/L control siRNA by incubation in the presence of Dharmafect 4 (Thermo Fisher Scientific) following the manufacturer’s instructions. The effect of the siRNA was analysed 60 hours after transfection, when silencing of EBP50 was effective.

### Statistics

Student *t*-test was used for comparisons. A *P* value of less than 0.05 was considered significant.

## Results

### The C-Terminus of ABCB4 ends by a PDZ-like Motif

**Fig 1:**
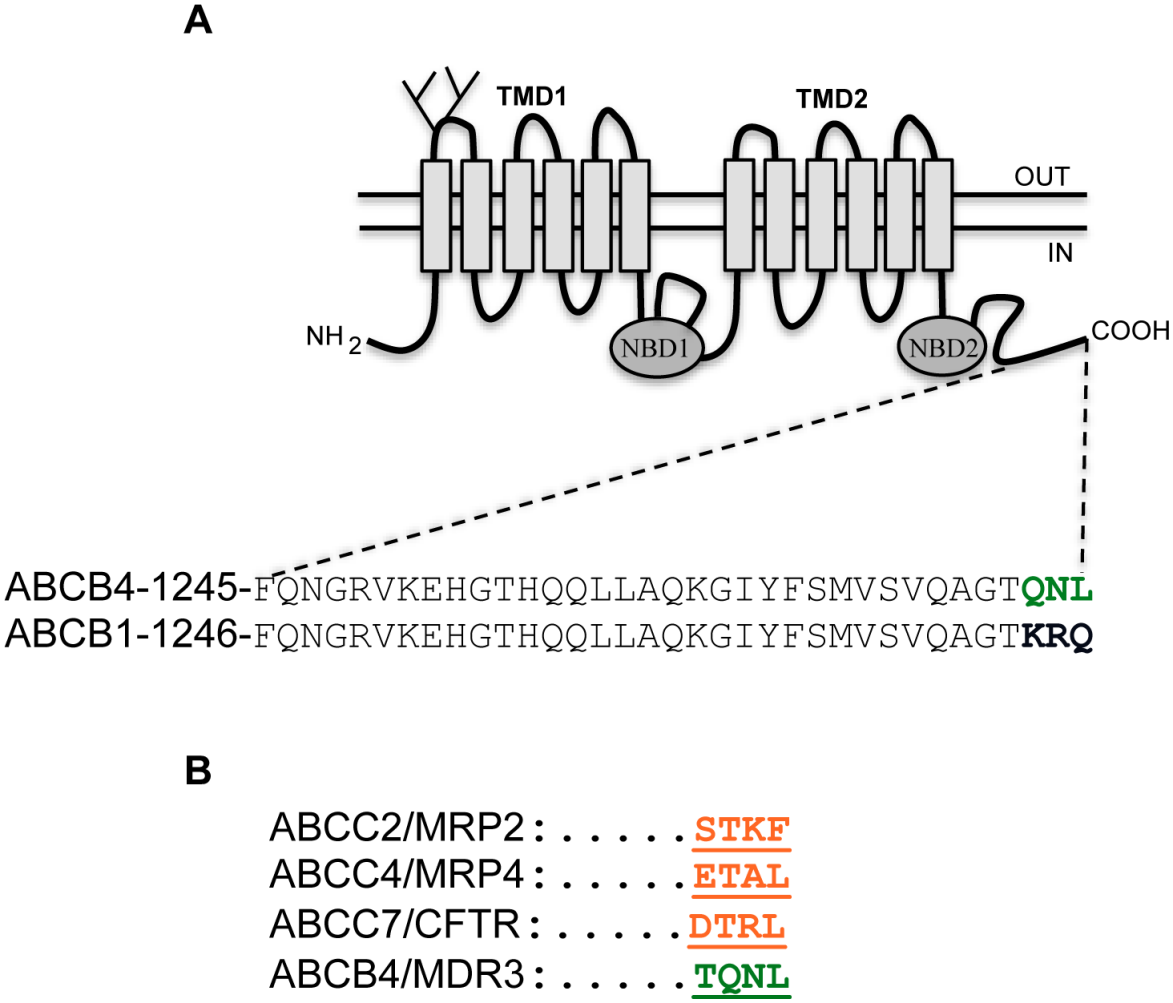
The C-terminus of ABCB4 ends by a PDZ-like motif. (A) Schematic representation of ABCB4. ABCB4 contains two transmembrane domains (TMD1 and TMD2) and two nucleotide binding domains (NBD1 and NBD2). The two glycosylation sites in the first extracellular loop are indicated. The amino acid sequence of the intracytoplasmic C-terminal domain of the human ABCB4 isoform A (NP_000434.1) and the human ABCB1 (NP_000918.2) are shown. Green letters indicate the PDZ-like motif of ABCB4. (B) A sequence alignment of known PDZ motifs found in ABC family members is shown. ABCC2, ABCC4 and ABCC7 display class I PDZ consensus motifs.

ABCB4 is highly homologous to the multidrug transporter ABCB1, sharing 77% identity at the amino acid level. The C-terminal region is remarkably identical, except for the last three amino acids. Alignment of the sequences of the last 35 amino acids of ABCB4 and ABCB1 is represented in Fig 1A. The sequences differ at the C-terminus, with ABCB4 ending by the sequence glutamine-asparagine-leucine (QNL), while the last three amino acids of ABCB1 are lysine-arginine-glutamine (KRQ). The QNL motif shows some similarity to the PDZ binding motifs identified in other ABC transporters (Fig 1B), especially the presence of a hydrophobic amino acid at the extreme C-terminus. These observations suggested that the QNL motif might interact with PDZ domain protein(s) and serve a specific function.

### Expression and Localization of ABCB4-wt and ABCB4-ΔQNL in HepG2 Cells

**Fig 2:**
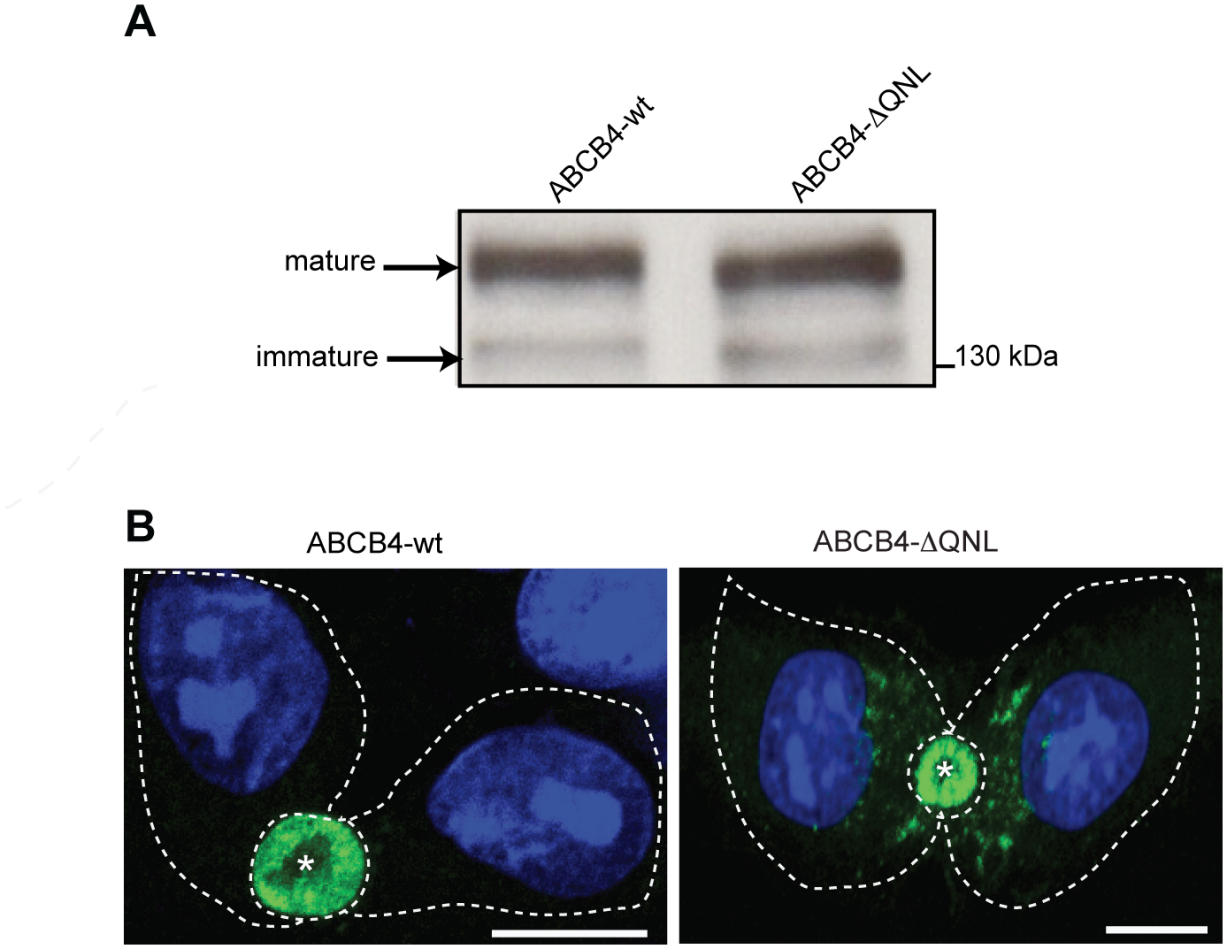
Expression and localization of ABCB4-wt and ABCB4-ΔQNL. (A) ABCB4 was detected by immunoblotting from cell lysates of HepG2 cells stably expressing ABCB4-wt or ABCB4-ΔQNL. (B) HepG2 cells stably expressing ABCB4-wt or ABCB4-ΔQNL were fixed with methanol/acetone, processed for immunofluorescence using the monoclonal P3II-26 antibody and Alexa 488-conjugated anti-mouse IgG and visualized by confocal microscopy. Nuclei were stained with DRAQ 5 (blue). Asterisks indicate bile canaliculi. Cell contours are indicated by dotted lines. Bars, 10 μm. Representative of three experiments.

To investigate the role of the PDZ-like motif, the QNL sequence was deleted by PCR-based site directed mutagenesis. The expression and subcellular localization of the generated mutant (ABCB4-ΔQNL) were assessed by western blotting and immunofluorescence, and compared to those of wild type ABCB4 (ABCB4-wt) after stable transfection in HepG2 cells. Immunoblotting showed that the electrophoretic behavior of ABCB4-ΔQNL was comparable to that of ABCB4-wt. Both proteins migrated as a mature and an immature form of *M*_r_ 160 and 140 kDa, respectively, as previously reported for ABCB4-wt [21] (Fig 2A). Immunofluorescence showed that ABCB4-wt and ABCB4-ΔQNL were both localized at the canalicular membrane (Fig 2B), although ABCB4-ΔQNL was occasionally detected intracellularly. These data indicated that the deletion of the QNL motif had little or no effect on the processing and membrane localization of ABCB4.

### Deletion of the PDZ-like Motif causes a Decrease of ABCB4 Protein Stability

**Fig 3:**
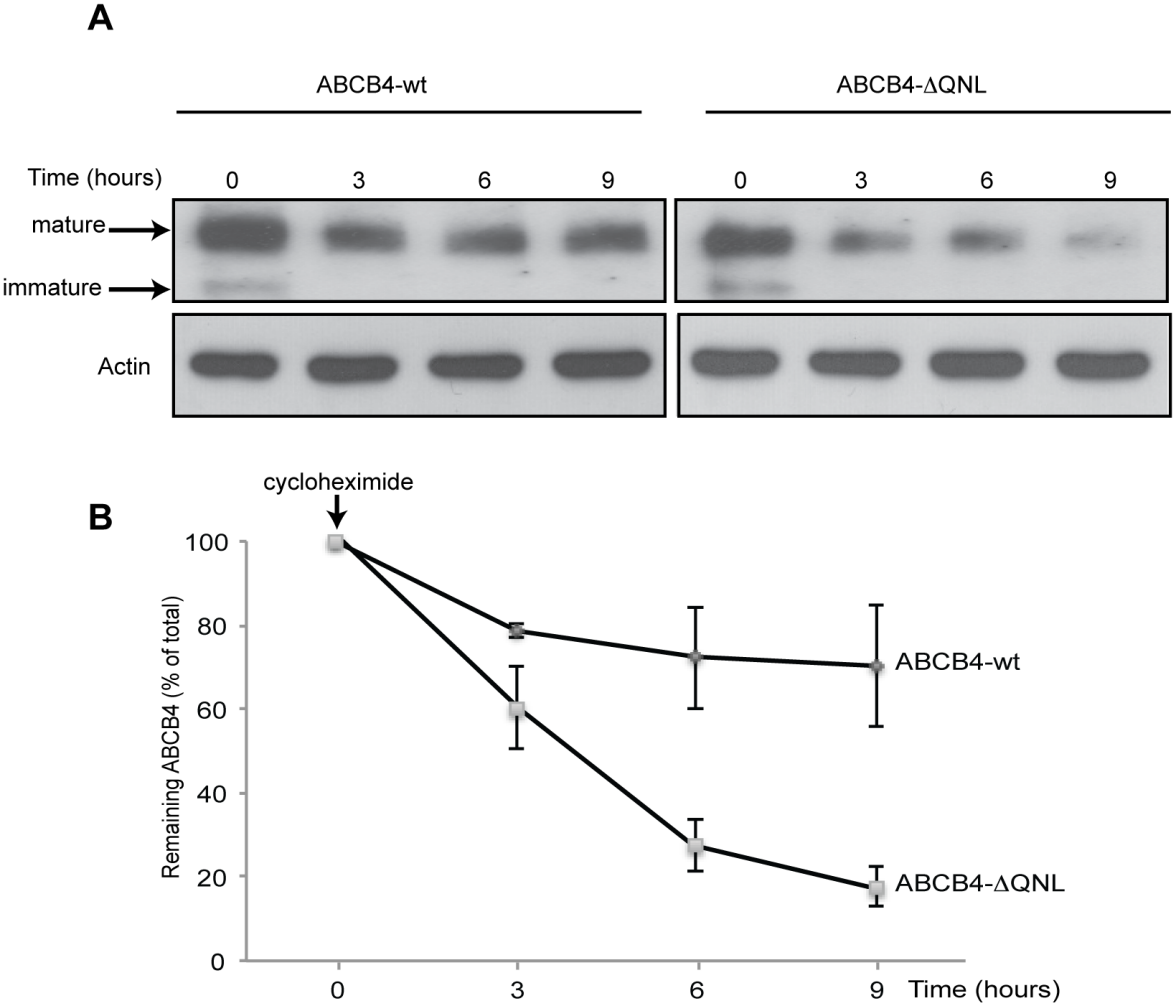
Stability of ABCB4-wt and ABCB4-ΔQNL. (A) Stability of ABCB4-wt or ABCB4-ΔQNL was analyzed in stably transfected HepG2, after inhibiting protein synthesis with cycloheximide (25 μg/mL). ABCB4 was detected at the indicated time points in cell lysates by immunoblotting, using equal amounts of total proteins per lane. (B) Amounts of ABCB4 were quantified from chase experiments. The amount of ABCB4 at time zero was considered as 100%. Remaining ABCB4 at later time points was expressed as percentage of time zero. Means (± SEM) of three independent experiments are shown. * P**<**0.05 at all points.

PDZ binding motifs play a role the stability of multiple ABC transporters at the cell surface [14, 15, 26]. Therefore, we tested whether ABCB4 stability would be altered as a result of deletion of the QNL motif. The stability of ABCB4-wt and that of ABCB4-ΔQNL were compared by analyzing the decay of ABCB4 protein expression after inhibition of protein synthesis with cycloheximide. HepG2 cells expressing either the wild type or the mutant ABCB4 protein were harvested at specific time points after cycloheximide addition and were evaluated by western blotting (Fig 3). Fig 3A shows a representative immunoblot. At time point 0, both mature and immature forms were observed for ABCB4-wt and ABCB4-ΔQNL. The immature form did not appear at later time points, consistent with inhibition of protein synthesis. After three hours, the amount of ABCB4-wt decreased by ~20% as compared to the time point 0, and thereafter this level remained constant until the 9-hour time point (Fig 3B). By contrast, the amount of ABCB4-ΔQNL continuously decreased under cycloheximide treatment (Fig 3A). After three hours, ABCB4-ΔQNL showed a ~40% decrease as compared to the time point 0. The reduction was more than 70% after 6 hours and 80% after 9 hours (Fig 3B). These results suggested that ABCB4-ΔQNL was degraded faster than ABCB4-wt.

### The QNL sequence of ABCB4 is a Plasma Membrane Retention Motif

Additional studies were conducted to determine whether deletion of the PDZ-like motif reduced the stability of ABCB4 by accelerating its endocytosis. We compared the kinetics of internalization of ABCB4-wt and of the truncated mutant ABCB4-ΔQNL, using an internalization assay, as previously described [25]. For these experiments, we generated an ABCB4 construct bearing a triple myc-tag (3xmyc) in the first extracellular loop. This allowed specific labeling of ABCB4 localized in the plasma membrane of non-permeabilized cells. In addition, since bile canaliculi of HepG2 cells are sealed and not accessible to antibodies in non-permeabilized conditions, we used HEK 293 cells that were transiently transfected with either 3xmyc-ABCB4-wt or 3xmyc-ABCB4-ΔQNL for the internalization assay. We checked by a functional assay [23], that addition of a triple myc-tag did not affect the phosphatidylcholine secretion activity (data not shown).

**Fig 4:**
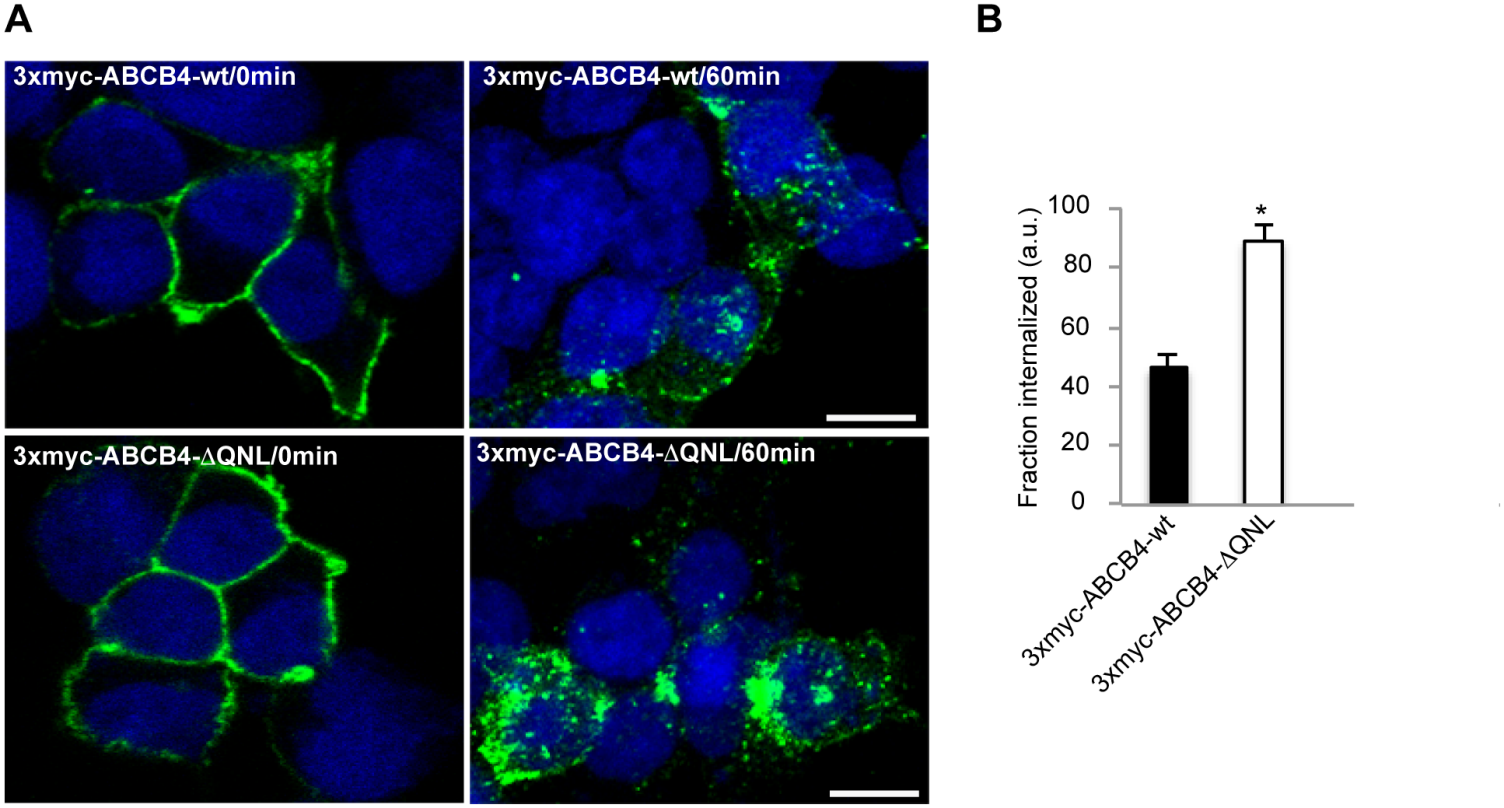
The internalization of ABCB4-ΔQNL is accelerated. (A) Transiently transfected HEK 293 cells were incubated for 30 minutes at 0°C with anti-myc antibody. After surface labeling, cells were incubated at 37°C for 60 minutes. At the end of the 37°C incubation, non-internalized antibody-antigen complexes were removed by acid washing. Cells were fixed, permeabilized and internalized antibodies were visualized with Alexa-Fluor 488-conjugated secondary antibodies and examined by confocal microscopy. Representative immunofluorescence images are shown. Nuclei were stained with DRAQ 5 (blue). Bars, 10 μm. (B) The amount of internalized ABCB4 was measured in cells transfected with 3xmyc-ABCB4-wt or 3xmyc-ABCB4-ΔQNL using ImageJ 1.41 software. Means (± SEM) of at least three independent experiments are shown. *P<0.001. a.u., arbitrary units.

HEK 293 cells expressing 3xmyc-ABCB4-wt- or 3xmyc-ABCB4-ΔQNL were incubated with an anti-c-myc antibody for 30 minutes at 0°C to allow binding of the antibody to the ABCB4 molecules expressed at the cell surface. Endocytosis was initiated by raising the temperature to 37°C. After 60 minutes, the cells were fixed, permeabilized and incubated with a fluorescently labeled secondary antibody. Before endocytosis, the antigen-antibody complexes were detected exclusively at the plasma membrane (Fig 4A, left panel). After 60 minutes at 37°C, both 3xmyc-ABCB4-wt and 3xmyc-ABCB4-ΔQNL were detected in intracellular vesicles, attesting that both proteins underwent endocytosis. However, the internalization rate of the truncated 3xmyc-ABCB4-ΔQNL was significantly higher than that of 3xmyc-ABCB4-wt (Fig 4A, right panel). Quantification of fluorescence showed that the rate of endocytosis of the truncated 3xmyc-ABCB4-ΔQNL was two-fold more rapid than that of 3xmyc-ABCB4-wt (Fig 4B). These data indicated that the PDZ-like motif QNL selectively retained ABCB4 at the plasma membrane.

### ABCB4 Colocalizes and Coimmunoprecipitates with EBP50

**Fig 5:**
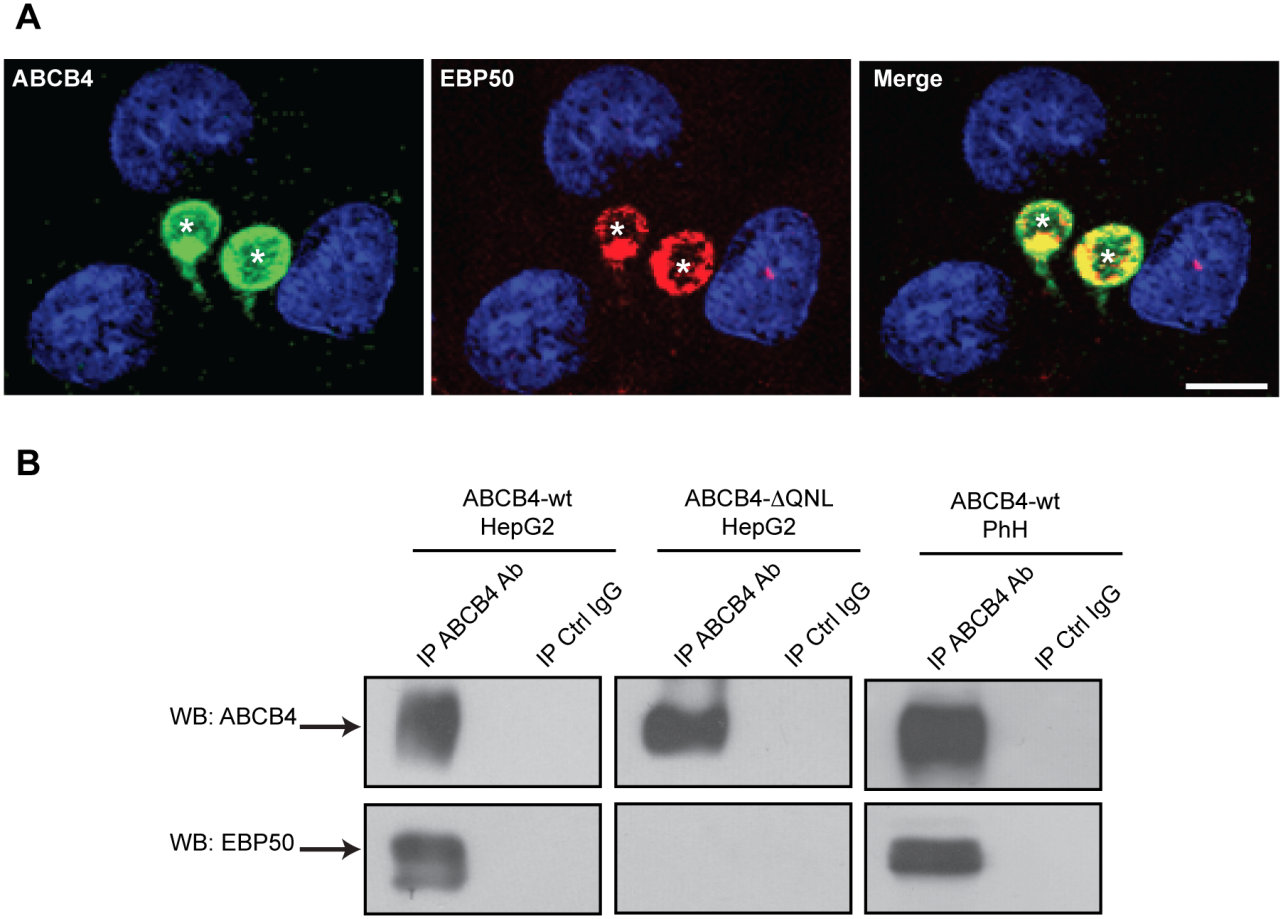
ABCB4 colocalizes and coimmunoprecipitates with EBP50. (A) ABCB4-wt-expressing HepG2 cells were fixed, permeabilized and stained with anti-ABCB4 antibody followed by anti-EBP50 antibody and then incubated with Alexa-Fluor-488-and 594-conjugated secondary antibodies and visualized by confocal microscopy. Nuclei were stained with DRAQ 5 (blue). Asterisks indicate bile canaliculi. Bar, 10 μm. (B) Cell lysates of HepG2 cells stably transfected with ABCB4-wt or ABCB4-ΔQNL or cell lysates of primary human hepatocytes (PhH) were incubated with anti-ABCB4 antibody or mouse imununoglobulin G (IgG) covalently linked to agarose beads. The coimmunoprecipitated complex was immunoblotted with anti-ABCB4 and anti-EBP50 antibodies.

To determine if EBP50 interacts with ABCB4, we examined if the two proteins colocalized and coimmunoprecipitated. By immunofluorescence, ABCB4 and EBP50 strongly colocalized at the canalicular membrane in ABCB4-wt-expressing HepG2 cells (Fig 5A). Colocalization was not altered by deletion of the QNL motif of ABCB4 (data not shown). Cell lysates from HepG2 cells that expressed either ABCB4-wt or ABCB4-ΔQNL were subjected to coimmunoprecipitation with an anti-ABCB4 antibody, followed by EBP50 detection by western blot. Fig 5B shows that EBP50 coprecipitated strongly with ABCB4-wt but not with the truncated mutant ABCB4-ΔQNL. Interaction between ABCB4 and EBP50 was also studied in primary human hepatocytes. As shown in Fig 5B, EBP50 coimmunoprecipitated with ABCB4-wt in freshly isolated human hepatocytes. These experiments showed that ABCB4 interacted with the PDZ protein EBP50 both *in vitro* and *in vivo*, and that the last three amino acids QNL were necessary for this interaction.

### Effect of Dominant-Negative Mutants of EBP50

**Fig 6:**
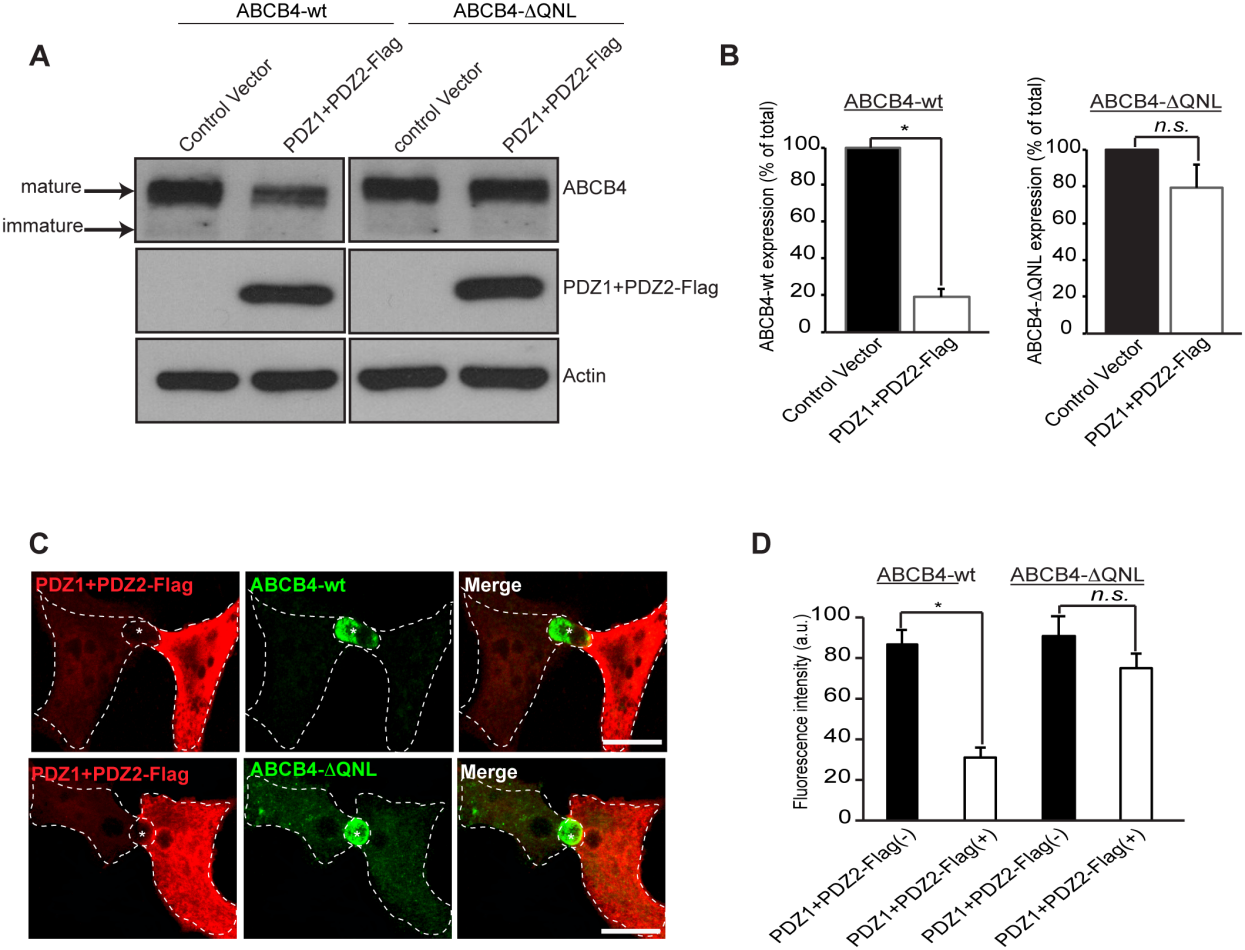
Effect of overexpression of the PDZ domains of EBP50. (A) ABCB4-wt- or ABCB4-ΔQNL-expressing HepG2 cells were transiently transfected with the Flag-tagged EBP50 PDZ domains (PDZ1+PDZ2-Flag) or with the empty vector-Flag (control vector). ABCB4 was detected by immunoblotting from cell lysates. (B) Amounts of ABCB4 were quantified from immunoblots by densitometry. ABCB4 levels were expressed as a percentage of total expression in HepG2 cell transfected with control vector. (C) ABCB4-wt- or ABCB4-ΔQNL-expressing HepG2 cells were transiently transfected with the Flag-tagged EBP50 PDZ domains (PDZ1+PDZ2-Flag). Cells were fixed, permeabilized and stained with the anti-Flag antibody followed by anti-ABCB4 antibody and then incubated with Alexa-Fluor-594-and 488-conjugated secondary antibodies and visualized by confocal microscopy. Representative immunofluorescence images are shown. Asterisks indicate bile canaliculi. Bars, 10 μm. (D) The amount of ABCB4 at the bile canaliculi was quantified in HepG2 transfected cells (PDZ1+PDZ2-Flag(+)) and compared to that observed in control adjacent non-transfected cells (PDZ1+PDZ2-Flag(-)). Means (± SEM) of at least three independent experiments are shown. *P<0.01 for ABCB4-wt; n.s., not significant; a.u., arbitrary units.

**Fig 7:**
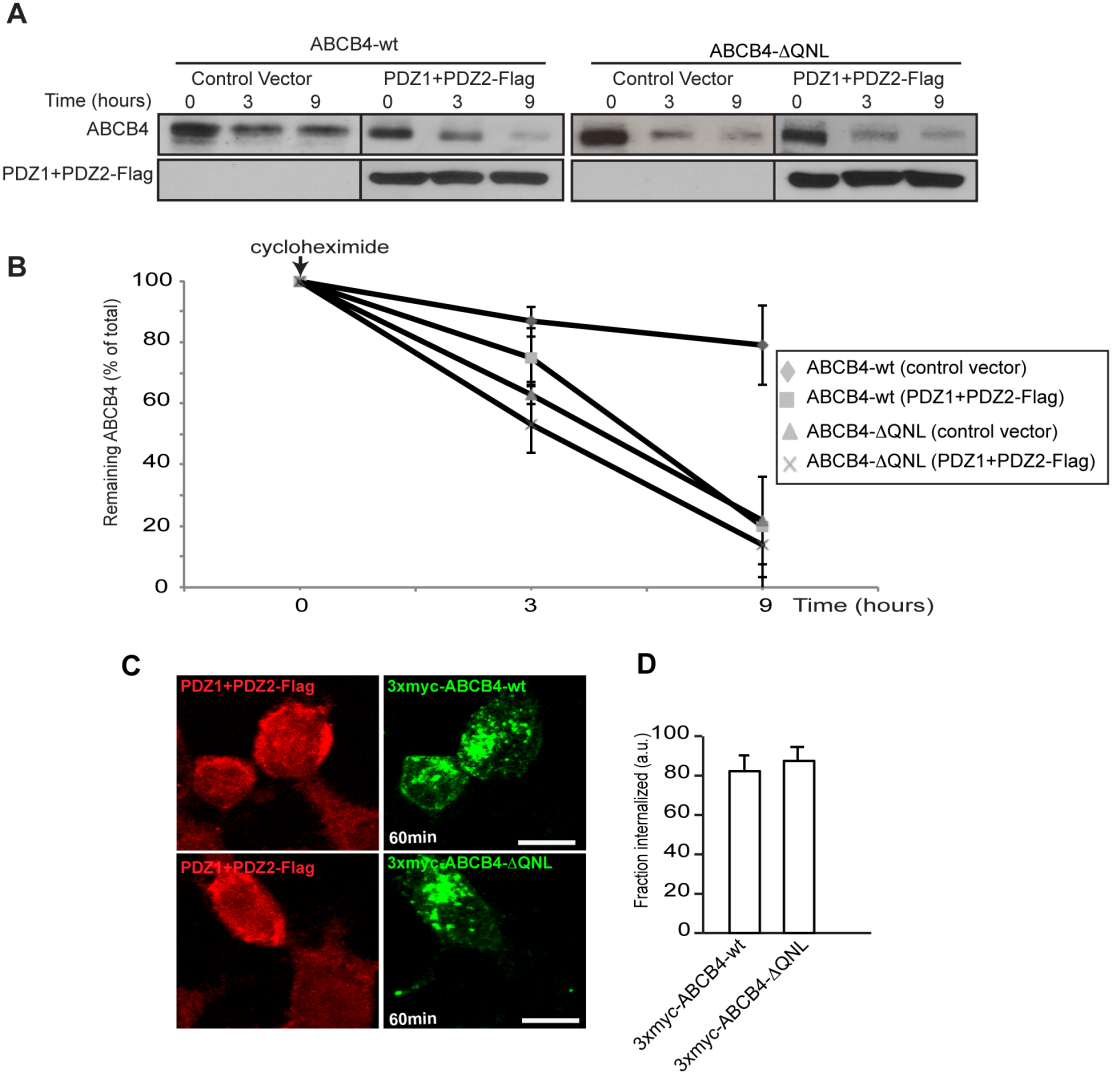
(A) Effect of overexpression of the PDZ domains of EBP50 on ABCB4 stability. ABCB4-wt- or ABCB4-ΔQNL-expressing HepG2 cells were transiently transfected with the Flag-tagged EBP50 PDZ domains (PDZ1+PDZ2-Flag) or with the empty vector-Flag (control vector). Stability of ABCB4-wt or ABCB4-ΔQNL was analyzed as described in Fig 3A. (B) Amounts of ABCB4 were quantified as described in Fig 3B. (C) Effect of overexpression of the PDZ domains of EBP50 on the membrane retention of ABCB4. The internalization assay was performed in HEK293 cells cotransfected with 3xmyc-ABCB4-wt or 3xmyc-ABCB4-ΔQNL as described in Fig 4A, but in the presence of PDZ1+PDZ2-Flag. (D) The amount of internalized ABCB4 was quantified as described in Fig 4B.

Taken together, the findings above suggested that the stabilization of ABCB4 at the plasma membrane could occur through interaction with the PDZ protein EBP50. In order to study the role of ABCB4-EBP50 interaction, we used a dominant-negative approach that consisted in overexpressing Flag-tagged EBP50 PDZ domains (PDZ1+PDZ2-Flag), without the ERM-binding domain [22]. This construct was transiently transfected in ABCB4-wt- and ABCB4-ΔQNL-expressing HepG2 cells and its effect was evaluated by western blotting. The expression of ABCB4-wt was strongly reduced in cells transfected with the PDZ interacting domains of EBP50 (PDZ1+PDZ2-Flag), whereas the expression of the truncated ABCB4-ΔQNL was poorly affected (Fig 6A). Quantification of western blots showed that the expression of ABCB4-wt was reduced down to ~20%, whereas the expression of ABCB4-ΔQNL was not significantly decreased (Fig 6B). The effect of overexpression of PDZ1+PDZ2-Flag was also studied by immunofluorescence. Fig 6C shows that the canalicular membrane expression of ABCB4-wt was strongly reduced in cells transfected with PDZ1+PDZ2-Flag as compared to adjacent non-transfected cells expressing ABCB4. Quantification of the fluorescence of ABCB4-wt at the bile canaliculi showed that the intensity was reduced down to ~35% in PDZ1+PDZ2-Flag transfected cells. In contrast, the canalicular localization of the truncated ABCB4-ΔQNL was not significantly decreased by the expression of the PDZ1+PDZ2-Flag construct (Fig 6C and 6D). We also examined the effect of overexpression of PDZ1+PDZ2-Flag on the protein stability and on the protein retention at the plasma membrane. We found that after 9 hours, the amount of ABCB4 decreased by ~80% in cells expressing the dominant negative mutants of EBP50, while the decrease was only ~20% in untransfected ABCB4-wt-expressing HepG2 cells. On the other hand, the degradation kinetics of the truncated ABCB4-ΔQNL mutant remained unchanged in cells expressing the dominant negative mutants (Fig 7A and 7B). Likewise, we found that in HEK293 cells, the internalization rate of 3xmyc-ABCB4-wt was similar to that of the truncated mutant 3xmyc-ABCB4-ΔQNL (Fig 7C and 7D, compare with Fig 4). Overall, these data provide evidence that the QNL motif interacts with the PDZ domains of EBP50 and regulates the stability of ABCB4 at the canalicular membrane.

### Effect of EBP50 Silencing on ABCB4 Expression

**Fig 8:**
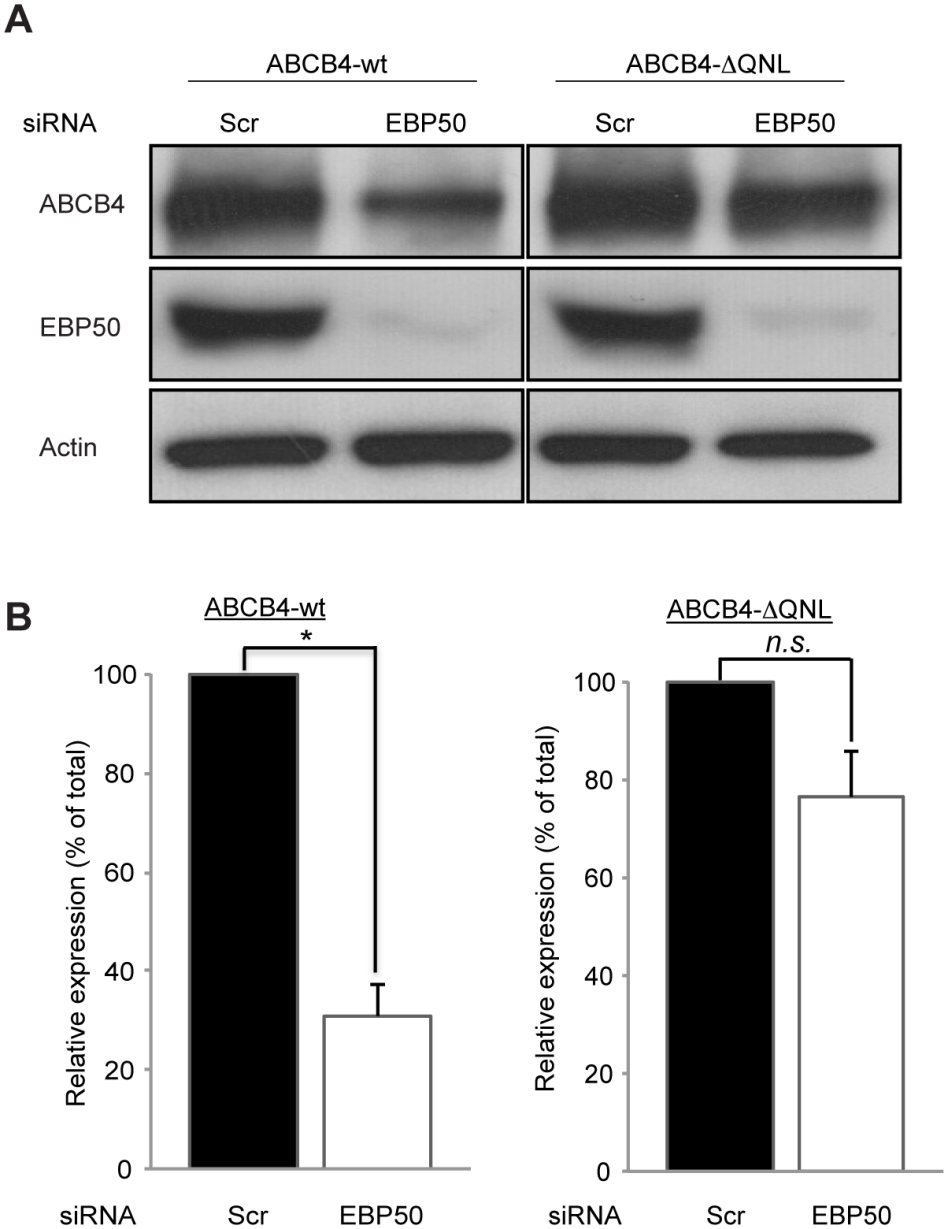
Effect of EBP50 silencing. (A) HepG2 cells expressing ABCB4-wt or ABCB4-ΔQNL were transfected with EBP50 siRNA or scramble siRNA (Scr). After 60 hours of transfection, cell lysates were subjected to western blot analysis. (B) Amounts of ABCB4 were quantified from immunoblots by densitometry. ABCB4 levels were expressed as a percentage of total expression in HepG2 cell transfected with scramble siRNA. Means (±SEM) of at least three independent experiments are shown. *P<0.05 for ABCB4-wt; n.s., not significant.

The functional association of EBP50 with ABCB4 was further evaluated after knocking-down of EBP50 by synthetic siRNA in ABCB4-wt- and ABCB4-ΔQNL-expressing HepG2 cells. As shown by western blot analysis (Fig 8A), 60 hours after siRNA transfection, the level of endogenous EBP50 protein was profoundly reduced in both ABCB4-wt and ABCB4-ΔQNL expressing cells. Decreased expression of EBP50 led to a marked reduction of ABCB4-wt down to ~30%, in HepG2 cells transfected with EBP50 siRNA relative to cells transfected with control scramble siRNA, whereas the expression of the truncated ABCB4-ΔQNL was not significantly affected (Fig 8A and 8B). By immunofluorescence, silencing of EBP50 also reduced the canalicular expression of ABCB4 (S1 Fig). These results provide further demonstration for the role of EBP50 in regulating the expression of ABCB4.

## Discussion

So far, very little was known about the mechanisms that regulate the cell surface expression of the phospholipid transporter ABCB4. Here we show that i) ABCB4 is retained at the canalicular plasma membrane by its C-terminal PDZ-like motif QNL, ii) ABCB4 through this motif interacts with the PDZ protein EBP50, iii) down-regulation of EBP50 or the expression of dominant-negative PDZ domains decrease the stability of ABCB4 protein.

The amino acid sequence at the C-terminus of ABCB4 is QNL and is highly conserved across species. Although it has some similarity with PDZ-binding motifs, this sequence does not fit with the consensus sequence [S/T]-xL, which is found in ABCC7/CFTR, in the ß_2_-adrenergic and purinergic P2Y1 receptors, and which is considered to be the optimal C-terminal motif for binding to the PDZ domains of EBP50 [27]. Nevertheless, we found that EBP50 coimmunoprecipitated with ABCB4, and that coimmunoprecipitation required the presence of the QNL motif. This motif closely resembles the non-canonical PDZ sequence QQL of the type II Na-coupled phosphate transporter-2c (Na/pPi-2c), which was shown to interact with EBP50/NHERF1 and NHERF3 [28, 29].

The mutant of ABCB4 missing the last three (QNL) amino acids was correctly expressed at the canalicular membrane of HepG2 cells, but its degradation was dramatically increased, as compared to wild type ABCB4. Instability of the mutant correlated with increased endocytosis at the plasma membrane. These results are consistent with those previously reported on the role of PDZ-binding motifs of other ABC transporters. Deletion of the PDZ motif from ABCC7/CFTR was found to reduce its half-life at the apical membrane of MDCK cells [30]. Removal of the PDZ-like motif GLV at the C-terminus of the basolateral transporter ABCC6/MRP6 also resulted in decreased steady-state levels of ABCC6/MRP6, decreased cell surface expression and stability, and mislocalization of the ABCC6/MRP6 protein in polarized cells [26]. The mechanism involved in the reduction of plasma membrane expression and increased degradation is not univocal. In the case of ABCC7/CFTR, the rate of endocytosis seemed unchanged after deletion of the PDZ-binding motif, but recycling of the protein to the plasma membrane was reduced [30]. Future studies will aim at clarifying the mechanisms by which degradation of the QNL-deleted ABCB4 mutant is accelerated.

The role of the QNL motif in the stability of ABCB4 at the canalicular membrane is very likely to occur through binding to EBP50. The expression of ABCB4, but not that of ABCB4-ΔQNL, was strongly reduced in the whole cell lysates and at the canalicular membrane of HepG2 cells, both after expression of a dominant negative mutant of EBP50 and after its invalidation by siRNA. Many transporters, channels, and receptors are known to interact with EBP50, and this interaction regulates their turnover by controlling their internalization and sorting to recycling endosomes or lysosomes. Disrupting the interaction between EBP50 and the ß_2_-adrenergic receptor leads to missorting and lysosomal degradation of the internalized receptor [31]. Similarly, down-regulation of EBP50 or disruption of its function by dominant-negative constructs increases internalization of the parathyroid hormone receptor and decreases membrane expression of this receptor [32].

EBP50 is an adaptor protein highly expressed in hepatocytes [12]. This is why, in the present study, we focused on the potential role of EBP50 on the membrane expression of ABCB4. However, several PDZ domain proteins may compete for binding to PDZ motifs. For example, the apical localization and the function of ABCC2/MRP2 have been shown to be regulated positively both by EBP50 and PDZK1/NHERF-3, another PDZ protein expressed in hepatocytes [15, 33]. Similarly, it has been shown that the activity of ABCC7/CFTR was dynamically regulated *via* a competitive balance between EBP50 and Shank2 [34]. Therefore, we cannot exclude that other PDZ proteins may contribute to the regulation of ABCB4 expression at the cell surface.

The regulation of secretion of bile constituents is directly linked to the mechanisms that regulate the trafficking, surface expression, and activity of their transporters. Expression of ABCC2/MRP2, which mediates the transport of conjugated organic anions, depends on its interaction with several PDZ domain-containing proteins, including EBP50. This interaction may be regulated by phosphorylation of a serine adjacent to the PDZ-binding motif in the transporter [35]. On the other hand, the bile salt transporter ABCB11/BSEP, which lacks a PDZ-binding motif, does not interact with EBP50 [15]. It has been shown that ABCB11 possesses a tyrosine motif in its cytoplasmic tail, which interacts with the adaptor protein AP2, and allows constitutive recycling of ABCB11 between the canalicular membrane and the sub-apical endosomal compartment [36, 37]. It is assumed that this mechanism provides a rapid response when bile acid secretion is needed [38]. ABCB4 does not contain such a motif, suggesting that it is probably not recycled constitutively. Our data provide evidence that the regulation of ABCB4 expression at the canalicular membrane is mediated by the C-terminal QNL motif, which constitutes an apical membrane retention motif *via* its interaction with EBP50. At the canalicular plasma membrane, the activity of ABCB4 would be regulated by phosphorylation of several residues [23]. These different mechanisms have to be tightly coordinated to ensure normal bile secretion.

## Supporting Information

S1 Fig Effect of EBP50 silencing on the canalicular expression of ABCB4. HepG2 cells expressing ABCB4-wt were transfected with EBP50 siRNA. After 60 hours of transfection, cells were fixed, permeabilized and stained with anti-ABCB4 antibody followed by anti-EBP50 antibody and then incubated with Alexa-Fluor-488-and 594-conjugated secondary antibodies and visualized by confocal microscopy. Nuclei were stained with DRAQ 5 (blue). Number 1 points to a cell in which EBP50 is down regulated and in which the canalicular expression of ABCB4 is reduced; number 2 points to a cell in which EBP50 is not down regulated and in which ABCB4 is highly expressed at the canalicular membrane. Asterisks indicate bile canaliculi. Bar, 10 μm.
(TIF)
