# Supplementary figures and images for: A PDZ-Like Motif in the Biliary Transporter ABCB4 Interacts with the Scaffold Protein EBP50 and Regulates ABCB4 Cell Surface Expression
Source: PLoS One. 2016 Jan 20;11(1):e0146962. doi: 10.1371/journal.pone.0146962 (PMC4720445; doi:10.1371/journal.pone.0146962)

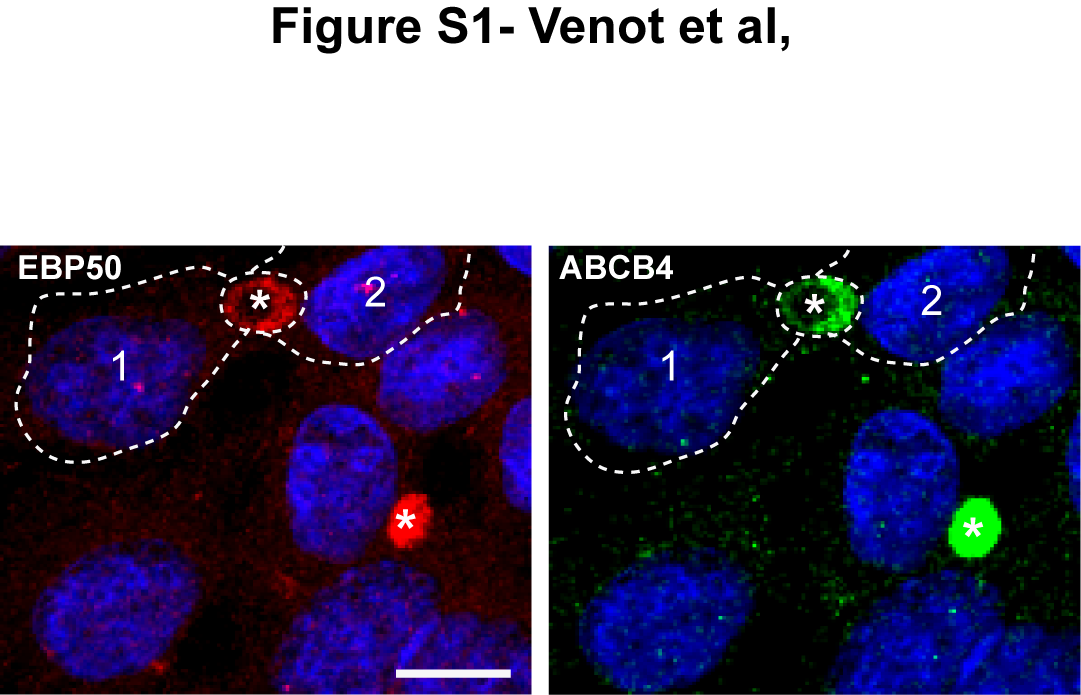

Supplement: S1 Fig — HepG2 cells expressing ABCB4-wt were transfected with EBP50 siRNA. After 60 hours of transfection, cells were fixed, permeabilized and stained with anti-ABCB4 antibody followed by anti-EBP50 antibody and then incubated with Alexa-Fluor-488-and 594-conjugated secondary antibodies and visualized by confocal microscopy. Nuclei were stained with DRAQ 5 (blue). Number 1 points to a cell in which EBP50 is down regulated and in which the canalicular expression of ABCB4 is reduced; number 2 points to a cell in which EBP50 is not down regulated and in which ABCB4 is highly expressed at the canalicular membrane. Asterisks indicate bile canaliculi. Bar, 10 μm. (TIF) [file pone.0146962.s001.tif]
